# Comparison of The Effects of A Positive Reappraisal Coping Intervention and Problem-Solving Skills Training on Depression during The Waiting Period of The Result of Intrauterine Insemination Treatment: A Randomized Control Trial

**DOI:** 10.22074/ijfs.2018.5155

**Published:** 2018-01-15

**Authors:** Marzieh Ghasemi Gojani, Masoumeh Kordi, Negar Asgharipour, Habibollah Esmaeili

**Affiliations:** 1 Student Research Committee, School of Nursing and Midwifery, Mashhad University of Medical Sciences, Mashhad, Iran; 2 Research Center for Evidence-Based Health Care, Department of Midwifery, School of Nursing and Midwifery, Mashhad University of Medical Sciences, Mashhad, Iran; 3 Research Center for Psychiatry and Behavioral Sciences, Department of Clinical Psychology, Mashhad University of Medical Sci- ences, Mashhad, Iran; 4 Research Center for Management and Social Factors Influencing Health, Department of Biostatistics, Faculty of Public Health, Mashhad University of Medical Sciences, Mashhad, Iran

**Keywords:** Artificial Insemination, Depression, Problem-Solving

## Abstract

**Background:**

The outcomes of fertility treatments are unpredictable, and levels of depressive symptoms increase in patients during the waiting period of the result of intrauterine insemination (IUI) treatment. The aim of this study was to compare the effects of a positive reappraisal coping intervention (PRCI) and problem-solving skills training (PSS) on depression during the waiting period of the result of IUI Treatment.

**Materials and Methods:**

This randomized control clinical trial was done among 108 women undergoing IUI treat- ment. In the control group, the women received routine care. In the PRCI group, women attended two training sessions and were asked to complete coping thoughts cards and fill out daily monitoring forms during the waiting period. In the PSS group, PSS were taught over three sessions. The depression was measured by the beck depression inventory.

**Results:**

On the 10^th^ day of the IUI waiting period, there were significant differences between the control group (21.42 ± 11.42) and the PSS group (12.52 ± 8.05) and PRCI groups (13.14 ± 9.7) (P<0.001), but no significant difference between the PRCI group and the PSS group.

**Conclusion:**

According to the results of this randomized control trial there is no difference between a PRCI and PSS on depression during the waiting period of the result of IUI treatment. This suggests that both interventions can be used to help infertile women combat depression during the waiting period of the result of fertility treatments (Registration number: IRCT2016020926490N1).

## Introduction

Infertility is defined as the failure to achieve a clini. cal pregnancy after 12 months or more of unprotectedsexual intercourse (1). Infertility can threaten the men. tal health of infertile couples. In a study by Peyvandiet al. (2), 62% of women who had been visiting in. fertility treatment had various degrees of depression: 27.5% suffered from mild depression, 25.5% moderate depression, and 9% severe depression. Intrauterineinsemination (IUI) treatment combined with ovulationinduction is usually considered the first-line treatmentfor many infertile couples and it is the most common ofthe treatment methods. Although with the advancement of science and new assisted reproductive techniquessuch as: *in vitro* fertilization (IVF), intra cytoplasmsperm injection (ICSI), and IUI, the hopes of infertilecouples have increased, these methods are expensiveand involve broad medical interventions and long periods of treatment (3).

The waiting period of the result of IUI treatment, refers to the time interval between the IUI operationand the time of the pregnancy test (4). This can beassociated with severe distress in individuals, the out. comes of fertility treatments are often unpredictableand the infertile women are not able to control or pre. dict the treatment outcome (5). According to a study by Osuna (6), the waiting period causes psychological reactions, stress, and anxiety in individuals as a result of their concerns about an event which will happen in the future, and which they are not able to predict or control. In a more recent study, Boivin and Lancastle (4) showed that the degree of anxiety and depression increased during the waiting period before fertility treatment.

One of the interventions designed to help people cope with the medical waiting period is the Positive Reappraisal Coping Intervention (PRCI). The PRCI is based on positive reappraisal coping strategies, and use of this intervention helps people emphasize the positive aspects of the situation (7). This technique has been used for the waiting period associated with IVF treatment, genetic tests, and recurrent miscarriage. In a study by Ockhuijsen et al. (8) use of positive coping thoughts cards led to an appreciation of the positive aspects of the situation and creation of a positive feeling in infertile women during the waiting period of the result of IVF treatment. Although research suggests an effect of the PRCI on positive feeling during the waiting period of the result of IVF treatment, there is disagreement about the effect of this intervention on negative emotions, anxiety, and depression in infertile women during waiting period (8, 9). It is also necessary to use other interventions to reduce depression during the period of waiting for fertility treatments.

Problem Solving Skills training (PSS) is a psychological intervention, which aims to help individuals adapt more effectively to stressful problems in life (10). Problem solving is one of the most important strategies in facing infertility (11). In a study by Kordi et al. (10), the severity of postpartum depression was significantly lower in the PSS group than in the control group. But no similar studies have been conducted on the effect of PSS on depression in infertile women. According to increased levels of depression during the waiting period and contradictions in the studies (8, 9). This randomized controlled trial was conducted with the aim of comparing the effects of a PRCI and PSS on depression during the waiting period of the result of IUI treatment in infertile women in the Milad Infertility Treatment Centre in Mashhad, Iran, during the years 2015 and 2016.

## Materials and Methods

This randomized control clinical trial involved 108 women, referred to the Milad Infertility Treatment Centre in Mashhad for IUI treatment. Sampling for the trial was undertaken after the research had been approved by the Ethics Committee of Mashhad University of Medical Sciences (Registration number: IRCT2016020926490N1) and consent obtained from the officials of Milad Infertility Treatment Centre. In order to prevent the dissemination of information between the groups were considered at three different times, so that after the completion of the sampling in a group, sampling was started in the other group. The manner of assignment was in this way that first the groups’ names were written on paper, then according to the draw, the first time interval was assigned to the control group, for the second period the PRCI group and for the third period PSS group was assigned. Available sampling method was applied in each group.

The sample size was calculated based on Cohen’s (1987) table, and considering a power of 80%, a confidence level of 95%, and an effect size of 70%, we determined 33 individuals to be required in each group. To take into account a 10% loss, we determined that 36 individuals were required for each group. Inclusion criteria for the study were: Iranian nationality, 18-40 years of age, ability to read and write, primary infertility, and obtaining a score less than 28 on the General Health Questionnaire (GHQ 28). Exclusion criteria for the study were: consumption of any psychoactive drug, occurrence of any stressful and unpleasant incidents over the past 6 months, suffering from medical illness, obtaining a depression score higher than 28 on the Beck Depression Inventory, cancellation of IUI treatment cycle, failure to participate in all training sessions, and unwillingness to continue cooperation in the research.

The instruments used in this trial included: a questionnaire on demographic and infertility-related information, the beck depression inventory, GHQ-28, daily monitoring forms, positive coping thoughts cards, and a checklist for implementing problem-solving skills. The questionnaire on demographic and infertility-related information consisted of questions about: age, level of education, employment status, duration of marriage, family income, duration of infertility, duration of treatment, number of times the participant had undergone IUI and IVF, cause of infertility, treatment seeking, expectancy of successful treatment, and the cost of treatment. The beck depression inventory contains 21 questions with answers scored between 0 and 3. The minimum possible score on the depression questionnaire is zero and the maximum is 63, classified as follows: minor depression 0-13, mild depression 14-19, moderate depression 20- 28, and severe depression 29-63.

The GHQ 28 is a questionnaire containing 28 questions that measure physical symptoms, anxiety, insomnia, social dysfunction, and severe depression. The answers are on a four-point Likert scale. The threshold score for this questionnaire is 28 and a score higher than 28 is a sign of susceptibility to mental disorders. The daily monitoring form was designed by Ockhuijsen et al. (8) to assess physical and mental changes in infertile women during the waiting period before fertility treatment. It consists of 46 questions related to the person’s emotions, physical symptoms, including symptoms related to anxiety and to the failure or success of treatment, coping strategies, person’s assessment during the period of waiting of the result of treatment and coping strategies during this waiting period. This form is a part of the PRCI and was completed each day by the PRCI group during the waiting period.

The positive coping thoughts card contains 10 statements based on the positive reappraisal coping strategy. The PRCI group repeated the positive thoughts at least twice a day during the waiting period. The validity of the qualitative content of this trial was assessed as follows: after the preparation and translation of the questionnaires (demographic data and information related to infertility, daily monitoring forms, positive coping thoughts cards, and check list of problem solving skills) under the supervision and guidance of professors in counseling, the questionnaires were reviewed by seven experts and professors from the Mashhad University of Medical Sciences. The final tools used incorporated the necessary revisions suggested by the experts. The reliability of the beck depression inventory (α=0.83), GHQ28 (α=0.83), and daily monitoring form (α=0.74) were ascertained using Cronbach’s alpha. Infertile women who visited the centre to plan their IUI treatment and who fulfilled the inclusion criteria for the study were recruited into the trial. PSS sessions and the PRCI sessions were performed by the researcher after confirmation of the researcher’s ability by a specialized consultant with a Ph.D. in clinical psychology.

In first session in the PSS group, which was on days 2-3 of the menstrual cycle, we discussed infertility and the IUI treatment process, the research objectives and how to conduct the sessions, and the role of using PSS in dealing with the problems of life. The participants were then asked to write a list of problems that they have had during the course of their IUI treatment and determine the most important issue. In the second session, on days 9-12 of the menstrual cycle, the participants were asked to suggest solutions to their problems using a brainstorming method which they were taught in the session and prepare a list of the solutions that came to mind. During the third session, on days 14-15 of the menstrual cycle, the participants discussed the disadvantages and advantages of implementing the solutions arrived at in the second session. Following the discussion they made a list of the disadvantages and advantages of implementing each of the solutions and chose the best solution. The participants were also taught how to evaluate the effectiveness of a solution and advised of the possibility of returning to the previous step in case a solution was deemed to be ineffective. They were asked to implement this PSS in dealing with their daily problems during the waiting period, and record their efforts in the checklist for implementing PSS.

The first session of the PRCI group was held on days 2-3 of the menstrual cycle. We discussed infertility, the IUI treatment process, the research objectives, types of coping strategy, and the positive reappraisal coping strategy. The second session was held on days 9-12 of the menstrual cycle. In this session we explained the ten statements on the positive coping thoughts card using examples and showed participants how to complete the daily monitoring form. The Participants were then asked to repeat the positive coping thoughts at least twice a day during waiting period. Control group participants received the center’s routine care, and presented themselves at Milad Infertility Treatment Center on days 2-3, 9-12, and 14-15 of the menstrual cycle to undergo an ultrasonography and determine any remedial measures for the IUI treatment. The beck depression inventory was completed by all the three groups on the 10^th^ day of the waiting period. Depression score means were compared between the three groups before the intervention and on the 10^th^ day of the waiting period.

### Statistical analysis

After collection and coding, the data were entered into the computer and analyzed using SPSS version 16, with P<0.05 considered statistically significant. The normality of the quantitative variables was determined using the Kolmogorov-Smirnov test. If the variables were normal parametric statistics were used, otherwise the non- parametric equivalent was used. Means, frequencies and standard deviations were used to describe the characteristics of the participants in each of the three groups. To compare depression between the three groups we used the ANOVA test and the paired t test for intra-group comparisons of depression if the data were normal. In the case of non-normal data the Kruskal-Wallis test and Wilcoxon tests were used.

### Results

**Fig.1:**
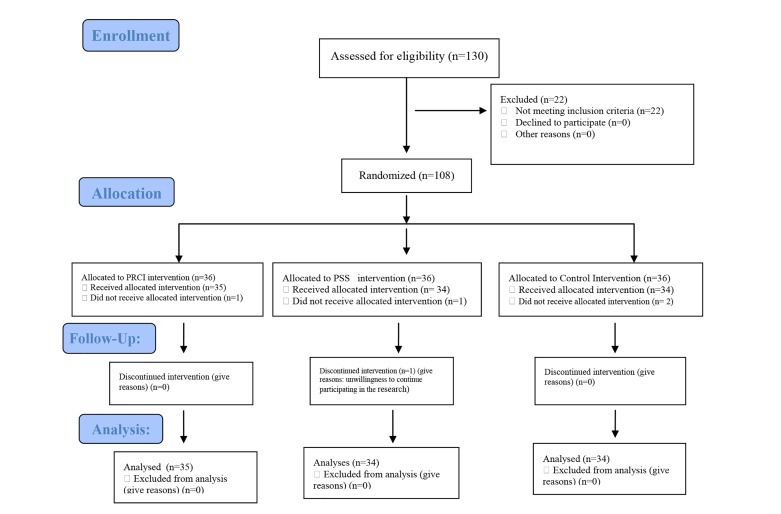
The stages of the intervention.

Data were obtained from 34 individuals in the control group, 34 individuals in the PSS group, and 35 individuals in the PRCI group. Two individuals in the control group and one individual in the PRCI group were excluded from the study due to the cancellation of their treatment program, and in the PSS group one individual was excluded from the study due to the cancellation of their treatment program, and one individual in the was excluded due to her unwillingness to continue participating in the research (Fig .1).

There was no significant difference between participants in the three groups in terms of level of education (P=0.853), the woman's occupation (P=0.364), cause of infertility (P=0.824), experience of using assisted reproductive techniques (P=0.410), and paying for the treatment (P = 0.392, Table 1). The mean GHQ28 score was 25.85 ± 3.93 in the control group, 25.28 ± 4.20 in the PSS three groups were homogeneous in terms of this variable (P=0.712, X^2^=0.68).

According to the paired t test and comparison of mean scores for depression in the two time intervals; before the intervention and on the 10^th^ day of the waiting period (a within-group comparison), the mean score for depression showed a significant decrease in the PSS group (P<0.001) and PRCI group (P=0.002), and a significant increase in the control group (P=0.007, Table 2).

**Table 1:** Descriptive statistics for infertile women undergoing IUI treatment in the three intervention groups: control, PSS, and PRCI group.


Variable		Group			Test results
		Control n(%)	PSS n(%)	PRCI n(%)		

Education level	Elementary school	7 (19.4)	4 (11.1)	2 (5.6)	X^2^=0.31
	Middle school	5 (13.9)	6 (16.7)	4 (11.1)	df=2
	High school	8 (22.2)	11 (30.6)	16 (44.4)	P=0.853^a^
	University	16 (44.5)	15 (41.6)	14 (38.9)	-
Woman's occupation	Housewife	30 (83.3)	30 (83.3)	25 (69.4)	ExactX^2^=4.42
	Office employee	5 (13.9)	5 (13.9)	6 (16.7)	P=0.364^b^
	Student	1 (2.8)	1 (2.8)	5 (12.9)	-
Cause of infertility	Male factor	4 (11.1)	4 (11.4)	5 (16.7)	-
	Female factor	10 (27.8)	14 (40)	16 (41.7)	-
	Joint factor	9 (25)	8 (20)	7 (19.4)	ExactX^2^=3.01
	Unknown factor	13 (36.1)	10 (28.6)	8 (22.2)	P=0.824^b^
Prior use of assisted reproductive techniques	None	17 (47.2)	20 (55.6)	19 (52.7)	-
	IUI	14 (38.9)	9 (25)	13 (36.1)	-
	IVF	0 (0.0)	0 (0.0)	1 (2.8)	ExactX^2^=0.39
	Other	5 (13.9)	7 (19.4)	3 (8.4)	P=0.410^b^
Paying for treatment	Not at all	2 (5.6)	4 (11.1)	2 (5.6)	-
	Low	6 (16.7)	8 (22.2)	4 (11.1)	X^2^=1.87
	Relatively high	13 (36.1)	12 (33.3)	15 (41.7)	df=2
	High	8 (22.2)	8 (22.2)	11 (30.6)	P=0.392^a^
	Very high	7 (19.4)	4 (11.1)	4 (11.1)	-


IUI; Intrauterine insemination, PSS; Problem-solving skills, PRCI; Positive reappraisal coping intervention, IVF; In vitro fertilization, a; Kruskal-Wallis, b; Fisher’s exact test, and df; degrees of freedom.

**Table 2:** Comparing the mean and standard deviation of depression scores before the intervention and on the tenth day of the waiting period of the result of IUI treatment in the control, PSS, and PRCI groups


Depression	Group			One-way ANOVA test result
	Control Mean ± SD	PSS Mean ± SD	PRCI Mean ± SD	

Before the intervention	17.38 ± 9.96	19.13 ± 8.67	18.55 ± 9.00	F=0.33 P=0.716
Tenth day of waiting period of IUI treatment	21.7 ± 11.42	12.52 ± 8.05	13.14 ± 9.7	F=9.29 P<0.001
Mean changes before the intervention and on the tenth day of waiting period of IUI treatment	-4.88 ± 9.46	5.94 ± 7.38	2.19 ± 10.17	F=15.87 P<0.001
Results of paired-t test	t=-2.867 P=0.007	t=4.286 P<0.001	t=3.278 P=0.002	


IUI; Intrauterine insemination, PSS; Problem-solving skills, PRCI; Positive reappraisal coping intervention, t; Statistics of the test, and F; Statistics of the test.

The ANOVA test results showed that there was no significant difference in mean depression score between the three groups (P=0.716) before the intervention. However, on the 10^th^ day of the waiting period of the result of IUI treatment, there were significant differences between the mean depression scores of the three groups (P<0.001). The results of the Tukey’s post hoc test showed that there were significant differences in mean depression scores between the control and the PSS group and also the control and the PRCI group (P=0.001, Table 2).

## Discussion

In the present study, depression increased in the control group during the waiting period of the result of IUI treatment. This is consistent with the results of studies conducted by Boivin and Lancastle (4) and Ockhuijsen et al. (8). Being in a medical waiting period causes psychological distress and increased levels of anxiety and depression in the infertile women (12). These feelings result from their concerns about an important event (the result of the pregnancy test) which will happen in the future and which (6) they are unable to change or control and about which there is little information through which they can predict the treatment outcome (4).

In our study, the mean depression score decreased in the PRCI group and PSS group. But, in a study by Ockhuijsen et al. (8), depression increased during the waiting period after IVF treatments in the PRCI group which is not consistent with the results of the present study. In this study the inclusion criteria for the study included being under IVF treatment and speaking the dutch language, but in the present study, those who obtained a score higher than 28 on the general health questionnaire and a depression score higher than 28 on the beck depression inventory, as well as cases who needed to be referred to a psychologist, were excluded.

In a study by Kordi et al. (10), after 5 sessions of PSS, the level of postpartum depression significantly decreased in the PSS group which is consistent with the results of the present study. Problem solving is an important coping strategy that enables an individual to appropriately control problematic situations (13, 14). PSS have an important role in mental and physical health, especially when people face unpleasant events and negative tensions in their lives (15). In a study by Talaei et al. (16), after 10 sessions of cognitive-behavioral therapy group training, the level of depression in infertile women significantly decreased in the cognitive-behavioral therapy group compared with controls which is consistent with the results of the present study.

Despite the fact that infertility treatments are stressful, infertile women expressed little desire to use the proposed psychological interventions. The reasons mentioned were as follows: fear of attending consultation sessions, loss of personal privacy, the cost of consultation, and ineffectiveness of the consultation process (17).

The PRCI is a new intervention based on positive reappraisal coping strategies for medical waiting periods, whose implementation does not need an in-person visit to the advisor, and which is affordable (5). Positive reappraisal coping strategies can help people adapt to unpredictable and long times, because positive reappraisal coping strategies lead to a reappraisal of the situation and emphasis on discovering the benefits and positive aspects during stressful conditions (8). The basis of this intervention is creating a positive thought through a cognitive process. The design of the PRCI is based on Lazarus and Folkman’s theory of stress and coping. In their study, Lazarus and Folkman came to the conclusion that positive emotions play the most important role in encouraging people to continue making efforts to cope with stressful situations (5, 7, 8, 12, 18).

Among the strengths of this study are the adaptability of the training sessions to the therapeutic program intended for the participants. A limitation of our study is that the trial was not double-blind, which introduces the possibility of observer bias. However, the fact that our findings are consistent with those of other studies in the field means that this is unlikely to have had a major impact on our results.

## Conclusion

As the results of this study found the impact of PRCI and PSS on depression during the waiting period of the result of IUI treatment were the same, it is suggested both interventions can be used, if facilities are available, to help infertile women reduce the depression generated by the waiting period and increase their adaptability.
